# FGCN: Image-Fused Point Cloud Semantic Segmentation with Fusion Graph Convolutional Network

**DOI:** 10.3390/s23198338

**Published:** 2023-10-09

**Authors:** Kun Zhang, Rui Chen, Zidong Peng, Yawei Zhu, Xiaohong Wang

**Affiliations:** 1 College of Information Science and Engineering, Hebei University of Science and Technology, Shijiazhuang 050018, China; zhangkun@hebust.edu.cn (K.Z.); cheerui@stu.hebust.edu.cn (R.C.); zhuyawei@stu.hebust.edu.cn (Y.Z.); 2 College of International Education, Guangxi University of Science and Technology, Liuzhou 545006, China

**Keywords:** point clouds, graph attention convolution, multi-modal data, multi-scale features, FGCN

## Abstract

In interpreting a scene for numerous applications, including autonomous driving and robotic navigation, semantic segmentation is crucial. Compared to single-modal data, multi-modal data allow us to extract a richer set of features, which is the benefit of improving segmentation accuracy and effect. We propose a point cloud semantic segmentation method, and a fusion graph convolutional network (FGCN) which extracts the semantic information of each point involved in the two-modal data of images and point clouds. The two-channel k-nearest neighbors (KNN) module of the FGCN was created to address the issue of the feature extraction’s poor efficiency by utilizing picture data. Notably, the FGCN utilizes the spatial attention mechanism to better distinguish more important features and fuses multi-scale features to enhance the generalization capability of the network and increase the accuracy of the semantic segmentation. In the experiment, a self-made semantic segmentation KITTI (SSKIT) dataset was made for the fusion effect. The mean intersection over union (MIoU) of the SSKIT can reach 88.06%. As well as the public datasets, the S3DIS showed that our method can enhance data features and outperform other methods: the MIoU of the S3DIS can reach up to 78.55%. The segmentation accuracy is significantly improved compared with the existing methods, which verifies the effectiveness of the improved algorithms.

## 1. Introduction

At present, semantic segmentation of point clouds has emerged as a highly significant research topic across various domains, including machine vision, artificial intelligence, photogrammetry, remote sensing, etc. [1,2,3,4]. Three-dimensional semantic segmentation is one of the crucial areas within the field.

Nowadays, the objective world is filled with various types of data, such as texts, images, sounds, point clouds, etc. However, in the field of computer vision, we give image and point cloud data special consideration. Point clouds provide rich geometric structures, while images contain texture information, both of which are beneficial for acquiring semantic information. Some early 3D semantic segmentation approaches only utilized single-modal data, which are point clouds. This approach was unable to differentiate between objects of the same category based on their colors or textures. As we all know, different modalities of data can provide more diverse and complementary information. Therefore, we have integrated point clouds with images, enabling the input data to encompass both depth and color information simultaneously [5,6,7].

**Figure 1:**
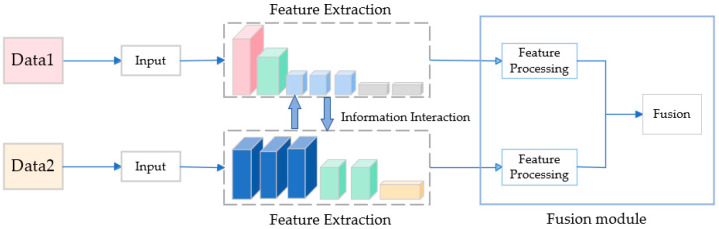
Fusion strategy based on complementary features process.

However, there are various methods to fuse different modalities of data. Common fusion strategies include fusion strategies based on discernible units (FSBDU), fusion strategies based on complementary features (FSBCF), fusion strategies based on target attributes (FSBTA), and fusion strategies based on multi-source decision (FSBMD) [8]. In our approach, we adopted the FSBCF method for data fusion. As shown in Figure 1, the original data were obtained from different modalities as inputs, features from each modality were extracted using separate feature extraction networks, the extracted features were processed, and the fusion was then performed.

A semantic segmentation network, called the FGCN, was suggested by us. The FGCN is intended to analyze the fused data from the point clouds and picture modalities and was inspired by FSBCF. The following can be said about our contribution:

(1) **Multi -modal topological map.** Data from the two modalities are combined using an image-guided structural representation of the topological map. The alignment algorithm of the image and point clouds is used to obtain multi-modal data. A two-channel KNN algorithm is used to obtain local information and construct a local topological structure map; (2) **Spatial attention mechanism**. For the extraction of more significant features, a spatial attention mechanism is employed. Average pooling and maximum pooling are performed in channel units and concatenate the pooled content. The spatial attention mechanism can assist the model in acquiring object features more effectively. It achieves this by introducing weights to the input data, and allowing the model to prioritize specific areas or objects of interest. (3) **Multi-modal SSKIT dataset.** An outdoor scene dataset called SSKIT was produced by us, and experimental validation was performed. The log_softmax classifier was added to the FGCN network and the semantic segmentation task was implemented using the SSKIT dataset and the S3DIS dataset, which validated the merits of the FGCN segmentation method proposed in this paper. The MIoU achieved on the SSKIT dataset was 88.06%; while on the S3DIS dataset, the MIoU reached 78.55%. Rigorous testing on the two reference datasets showed that our technique performs better than others. When this paper is published, we would like to open the dataset.

## 2. Related Work

In this section, we review point cloud semantic segmentation works. Three categories of segmentation methods are concerned with the perspective of different modal data. In the early days, semantic segmentation was implemented on the single-modal data of point clouds. Subsequently, for the use of semantic information in images, the two-modal data of images and point clouds were applied in segmentation mainly using fusion strategy. In this paper, the fusion strategies fall into two major categories: point clouds projected onto the image methods, and image-guided point clouds featuring learning methods.

### 2.1. Point Clouds-Only Methods

Many semantic segmentation methods use point cloud data exclusively. These methods can be roughly categorized into three groups. The first category is based on projection methods [9], which involve transforming 3D point clouds into 2D grids using techniques like spherical [10] or bird’s-eye view [11] projections. This approach enables the utilization of powerful existing 2D segmentation models [12]. However, due to this transformation, the point clouds’ geometric properties in three dimensions can be degraded. The second category involves the direct processing of original 3D point clouds [13,14]. Methods in this group often summarize the input point clouds using a set of sparse key points. PointNet [15] and PointNet++ [16] are representative examples of this approach. The third category comprises voxelization techniques [17,18], which involve grouping points within the point clouds into clusters that are treated as individual units. The original point clouds are processed and transformed into a voxel grid.

### 2.2. Point Clouds Are Projected onto Image Methods

Point clouds have rich geometry features, and images have rich texture features. The spatial properties of 3D point clouds are merged into 2D images to enhance feature representation and provide the network the ability to learn object distance information. D. K. Kim et al. [19] proposed a framework for projecting the features of 3D point clouds in 2D images to achieve robust pixel semantic segmentation of off-road terrain. The multi-scale data of the 3D point clouds and the image were trained separately by F. Yang et al. [20]. Multi-scale sparse point clouds are projected onto the 2D images, allowing the network to perform better feature learning. The final road detection model can eliminate shadows, curbs, and other interferences in complex scenes. To achieve high-accuracy road detection, L. Caltagirone et al. [21] projected a 3D point cloud onto the 2D plane of the camera and learned dense 2D image features with spatially encoded information by upsampling. Ye Chao et al. [22] improved the accuracy of semantic segmentation by transforming scattered point cloud features into densely distributed 2D spherical features through spherical mapping and spatial modules. Occlusion is the source of low-target detection accuracy; H. Wang et al. [23] employed grid projection and the fusion method to find obstacles in the 3D point cloud data. Compared with YOLO, the average accuracy of the road detection was improved by 17%.

### 2.3. Image-Guided Point Clouds Feature Learning Method

The precision of image feature learning networks fall short of requirements, which can be overcome using the image-guided point cloud feature learning approach. A 3D point cloud guided image fusion module was proposed by T. Huang et al. [24] to determine the semantic features of images. This module adaptively enhanced the characteristics of point cloud data and achieved good results on KITTI and SUN-RGBD datasets. A. S. Mohamed et al. [25] studied pre-fusion, mid-fusion, and post-fusion of images and point clouds. It was verified that mid-term fusion enabled better feature learning and is most effective on semantic segmentation tasks. V. Poliyapram et al. [26] proposed a multi-modal feature learning method that can act directly on the end-to-end structure of the point clouds. Image features are integrated into the 3D point cloud data to improve the modeling effect. L. Deng et al. [27] used a superpoint approach for the feature learning of images and point clouds, which showed some improvements in the semantic segmentation task. For the feature-level fusion of 2D feature maps and 3D point cloud aerial views, J. H. Yoo et al. [28] transformed 2D map features into smooth spatial features and achieved STOA performance in the KITTI benchmark. The SqueezeSeg method proposed by K. El Madawi et al. [29] added image RGB information to point clouds and showed good performance in the field of semantic segmentation. The network verified that adding RGB information leads to better feature learning, and there was almost no change in the time complexity of the network.

## 3. Methodology

We employ an image-guided point cloud feature learning method to fuse the original data. To construct a multi-modal topological map that can contain both point clouds and image information [30], 2D image data and 3D point cloud data are jointly feature represented $F_{mp}$.

Two-dimensional images are acquired by the camera and adjusted by the camera’s internal parameters. The image is rich in texture information, so the image $m_{i}$ can consist of pixel points $\left(u,v\right)$ with three channels of r, g, and b for each pixel point. The 3D point cloud data acquired by the Velodyne HDL-64E rotating 3D laser scanner acquisition consists of the spatial coordinates x, y, z, and the intensity information i. The image and point cloud representation are shown in the Equation:

(1) $$\left\{\begin{array}{l} m_{i}=(R(u,v),G(u,v),B(u,v))\\ p_{i}=\left\{\left.p_{i}\right|\right.x_{i},y_{i},z_{i},\left.i_{i}\right\} \end{array}\right.,$$

where $m_{i}$ is the feature representation of the image and $p_{i}$ is the feature representation of the point clouds.$\left(u,v\right)$ represents the pixel coordinates. *R*, *G*, and *B* are the color channel values, $x_{i}$, $y_{i}$ and $z_{i}$ are the point transport coordinates, and $i_{i}$ is the point cloud reflection intensity information.

**Figure 2:**
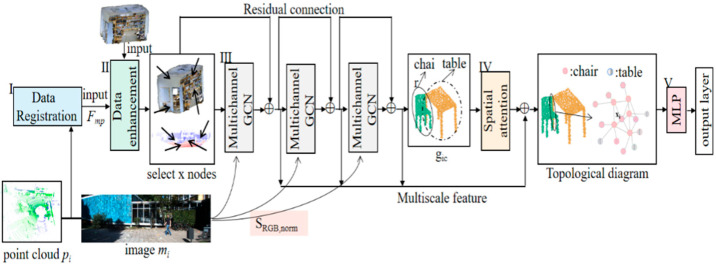
FGCN network structure. I–V are the operating procedures of the FGCN network.

A graph convolutional network named FGCN is proposed. As shown in Figure 2, the FGCN consists of five core modules, which are data alignment, data enhancement, building topological maps, spatial attention mechanism, and multi-layer perceptron.

The 3D point clouds are registered with the 2D image data using camera and radar parameters. A unique pixel point correspondence is found for each point cloud and the color information is added to the point cloud to obtain $p_{i}$, $p_{i}=\left\{\left.p_{i}\right|\right.x_{i},y_{i},z_{i},r_{i},g_{i},\left.b_{i}\right\}$. Data enhancement is performed by Module II. The multi-channel graph convolution network of Module III constructs an image-guided topological graph structure, and the graph is constructed by combining chromaticity values $S_{RGB,norm}$. The feature learning is enhanced by adding residual connections to the multilayer graph convolution to obtain the local feature map, $g_{ic}$. The data features are learned after the spatial attention of Module IV and then aggregated into a multi-mode topological diagram. The prediction labels for each point are obtained after the Module V MLP and output layers.

### 3.1. Image-Guided Multi-Channel Graph Convolution

In the field of computer vision, spatial graph convolution networks for feature extraction can optimize the problems of inadequate local information extraction and limited regional information merging. Therefore, this paper uses graph convolutional networks. It is necessary to convert 3D point clouds into vectors before entering them into the network. A sequence of points $\left\{x_{1},x_{2},x_{3},\ldots x_{n}\right\}$ is written as a multidimensional tensor using the mapping function γ, as shown in the Equation:

(2) $$f\left(x_{1},x_{2},x_{3},\ldots x_{n}\right)=\overset{n}{\underset{i=1}{\gamma }}\left(x_{i}\right),$$

where γ is the mapping function.

Since the KNN algorithm focuses more on the distance between nodes and the graph convolution focuses more on the edge information, we use images to guide the graph creation. The image’s color information is extracted as a compositional attribute, and the color information is added to the composition after the KNN aggregates the neighborhood features. The RGB feature set can be represented as $S_{RGB}$, $S_{RGB}=\left\{R,G,B\right\}$. In the multichannel KNN module, the RGB features are processed as a chromaticity-valued feature set $S_{RGB,norm}$ to represent the color features, as shown in the Equation:

(3) $$S_{RGB,norm}=\left\{\frac{R}{R+G+B},\frac{G}{R+G+B},\frac{B}{R+G+B}\right\}=\left\{r^{\prime },g^{\prime },b^{\prime }\right\},$$

where $S_{RGB,norm}$ is the chromaticity value of the color image, and $r^{\prime }$, $g^{\prime }$ and $b^{\prime }$ are the values after the color channel operation.

Relying on color invariance in the form of normalized colors, this color is insensitive to the illumination intensity. The new point cloud representation is shown in the Equation:

(4) $$p_{i}=\left\{p_{i}\left|x_{i},y_{i},z_{i},\right.r^{\prime },g^{\prime },b^{\prime }\right\}.$$

**Figure 3:**
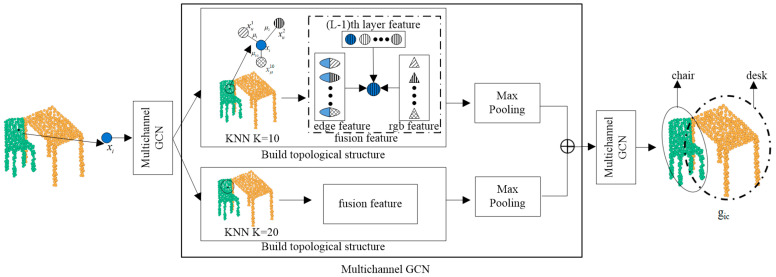
Image-guided graph structure building.

The process of building the graph structure is shown in Figure 3, where the image-guided topological graph structure is built around the input sequence of central nodes. The two-channel KNN structure extracts the features of the central node $x_{i}$ and uses different K values to ensure that the local features are fully learned.

As shown in Figure 3, The topological map structure aggregates the central node information, edge information, color information, and node information aggregated in the previous layer. The spatial and color features of each point cloud are fully utilized to speed up learning. The aggregation process of neighboring node features ${x^{\prime }}_{ic}$ of node $x_{i}$ is shown in the Equation:

(5) $$\left\{\begin{array}{l} x_{i}{}^{\prime }=\sum_{j:(i,j)\in \varepsilon }h_{\Theta }(x_{i},x_{j})\\ {x^{\prime }}_{ic}=\Lambda_{\Theta }({x^{\prime }}_{i},x_{i}^{c}) \end{array}\right.,$$

where $\left(x_{i},x_{j}\right)$ is the edge features between nodes $x_{i}$ and $x_{j}$. $h_{\Theta }$$\Lambda_{\Theta }$ are nonlinear functions with learnable parameters of the layer. $x_{i}{}^{\prime }$ and $x_{i}^{c}$ are edge features and color features of $x_{i}$. Respectively, ${x^{\prime }}_{ic}$ is a node of aggregated color features, where the label c represents the color. The node attribute feature ${\vec{x}}_{i}=\left\{x,y,z,r^{\prime },g^{\prime },b^{\prime },i\right\}$ forms the local feature graph $r^{\prime }$, $g^{\prime }$, and $b^{\prime }$ attribute information can enhance the graph structure building.

After constructing the graph structure, the edge features of the center point and the selected point are fused to achieve the aggregation of local and global information within the neighborhood graph. The *l*th layer features of ${x^{\prime }}_{ic}$ after fusion are shown in the Equation:

(6) $$h_{ij}^{l}=\Lambda_{\Theta }({x^{\prime }}_{ic},{x^{\prime }}_{jc}-{x^{\prime }}_{ic}).$$

After aggregating the features, max-pooling is used to filter the features. The max-pooling operation replaces an area’s output with the highest value found in the nearby rectangular region at a position. The max-pooling operation is shown in the Equation:

(7) $${x^{\prime \prime }}_{i}=\underset{j:\left(i,j\right)\in E}{\max}h_{ij}^{l}.$$

Inspired by the jump connection network, this paper connects the local features with residuals after computing them. To improve graph structure perception using nearby nodes, the input and output of the current layer are used as the input of the following layer. The residual connectivity is shown in the Equation:

(8) $$h_{i+1}=h_{i}+ℑ(h_{i},W_{i}),$$

where $h_{i}$ is the feature at layer *i*, $h_{i+1}$ is the feature at layer *i +* 1, $W_{i}$ is the convolution operation at layer *i*, and ℑ is the residual function.

The FGCN uses a three-layer graph convolution structure, and the central node $x_{i}$ aggregation is the last layer of features. Taking the *l*th layer graph convolution as an example, the nodes are first embedded, and the neighborhood information of the nodes is aggregated using the two-channel KNN algorithm. All of the neighborhood node information, edge information between nodes, and aggregated node information from the *l*-1th layer are contained in the *l*th layer features. To create a local neighborhood feature graph with color information, the layer *l-1* feature graph is first enhanced with color information. The features from the previous layers, color features, and edge features are all simultaneously aggregated when the layer 1 graph convolution process is carried out. The local feature map $g_{ic}$ is created by combining the features of all the nodes in layer 3, as shown in the Equation:

(9) $$\left\{\begin{array}{l} h_{v}^{l-1}=f\left(w^{l-1}x_{i}+\sum_{u\in N\left(v\right)}\mu_{v}x_{u}\right)\\ h_{v}^{l}=f\left(w^{l}x_{j}+\sum_{i=1}^{l}\sum_{u\in N\left(v\right)}\eta^{l}h_{u}^{\left(l-1\right)}\right) \end{array}\right.,$$

it is the aggregation mode of layer *l*-*1* features and layer *l* features of graph convolution. Where $h_{v}^{l-1}$ is the feature of layer *l*-*1*, w,η,μ are the learnable parameters, $x_{u}$ is one of the neighborhood nodes. $h_{v}^{l}$ is the feature of layer *l*, and $N\left(v\right)$ is the set of neighborhood nodes of node *v*.

The sense field is expanded using the structure of multi-layer graph convolution. All node features are aggregated to the centroid, which eventually forms a local neighborhood graph $g_{ic}$.

### 3.2. Spatial Attention Module

The multi-channel graph convolutional network learns local features, and use of the spatial attention mechanism may concentrate on crucial areas in the local features and handle the scene’s critical elements.

**Figure 4:**
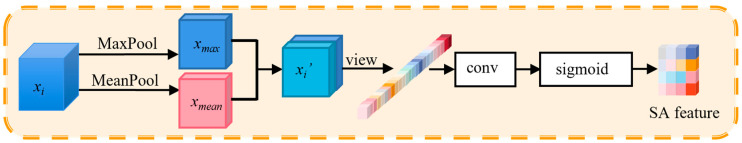
Spatial attention module.

As shown in Figure 4, first, each channel of the feature $x_{i}$ is subjected to mean operation and max operation respectively. The $x_{mean}$ obtained by the mean operation contains the basic shape and overall distribution of the features in the scene, and the $x_{\max}$ obtained by the max operation contains the salient features of each object. Then, $x_{\max}$ and $x_{mean}$ are stitched to get $x_{i}{}^{\prime }$, and the attention weights are obtained by convolution and pooling layers.

Spatial attention is shown in the Equation:

(10) $$x_{i}{}^{\prime }=\sigma \left(f^{7\times 7}\left(\left[MaxPool\left(x_{i}\right);MeanPool\left(x_{i}\right)\right]\right)\right)=\sigma \left(f^{7\times 7}\left(\left[x_{\max};x_{mean}\right]\right)\right),$$

where MaxPool is the maximum pooling function and $x_{\max}$ is the maximum pooling result of the channel. MeanPool is the average pooling and $x_{mean}$ is the average pooling result of the channel. $f^{7x7}$ is the convolution function. σ is the nonlinear activation function.

Spatial attention allows for the analysis of the details between objects at a local scale and uses residual structures to accelerate feature transfer. This module extracts attention information and makes the weights non-negative using sigmoid. The information about the same object in the immediate area can be filtered using the spatial attention module, while the information about interference can be ignored. The spatial attention mechanism can concentrate on essential information within the data, aiding the model to acquire pertinent features more efficiently and enhancing the model’s performance. The spatial attention mechanism plays a crucial role in enabling the model to better manage noise and interference. In our ablation study, it becomes evident that the removal of the spatial attention module leads to a significant drop in both the mean accuracy (MAcc) and overall accuracy (OAcc) of the model, indicating that point cloud noise has a detrimental impact on segmentation performance. Additionally, we noted that when the point cloud becomes excessively sparse, the spatial attention mechanism’s ability to handle noise diminishes.

### 3.3. Output Layer

**Figure 5:**
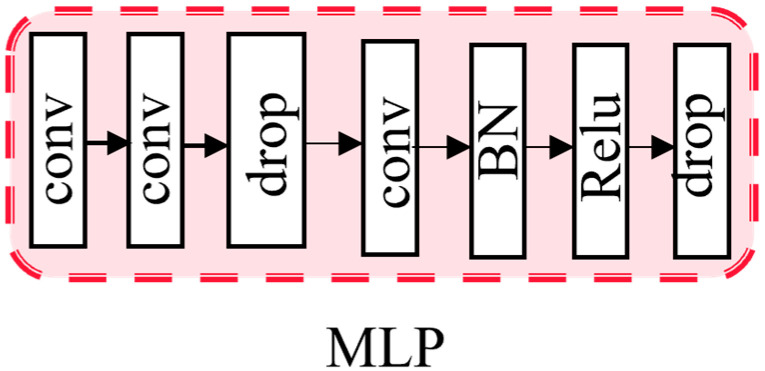
MLP module.

After obtaining spatial attention weights, deeper and more semantic information is multi-scale fused with shallow features. As shown in Figure 5, MLP is used to help the network converge. A nonlinear factor is introduced through the activation function $f\left(x_{i}\right)$. $f\left(x_{i}\right)=\max\left(0,x_{i}\right)$. The nonlinear activation function Relu is used in MLP, which allows the neural network to learn and represent complex linear relationships. Batch normalization [31] is also used, which helps to alleviate the vanishing gradient problem and accelerates the convergence of the model by normalizing the input of each layer to have zero mean and unit variance.

After the convolution operation, the labels and categories are processed into a dictionary form for the statistics and output of the results; the Equation is as follows:

(11) $$\overset{i=c}{\underset{i=1}{f_{dic}}}\left(num_{i},l_{i}\right)=\left\{num_{1}:l_{1},num_{2}:l_{2},\ldots num_{c}:l_{c},\right\},$$

where $num_{i}$ is the category, $l_{i}$ is the label, and $f_{dic}$ is the dictionary.

**Figure 6:**
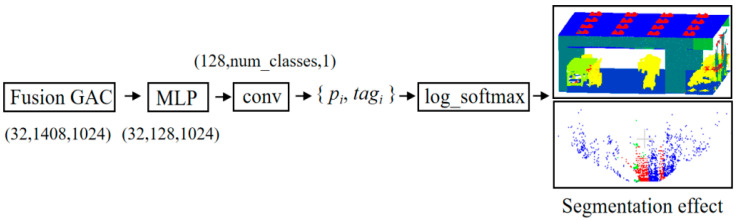
Output layer.

As shown in Figure 6, the Softmax function makes one prediction for all categories of each point cloud $x_{k}$. These predictions are given different weight values, and the prediction with the largest weight is taken as the final result. The use of log_softmax can prevent data overflow and thus improve data stability. Speeding up the derivation in the training process makes the backpropagation operation faster, as is shown in the Equation:

(12) $$\left\{\begin{array}{l} \sigma {\left(x\right)}_{j}=\frac{e^{x_{j}}}{\sum_{k=1}^{n}e^{x_{k}}}\\ log\_Softmax=log_{e}\left|\sigma {\left(x\right)}_{j}\right| \end{array}\right..$$

## 4. Results

### 4.1. Experimental Data and Environment

#### 4.1.1. Datasets

**Figure 7:**
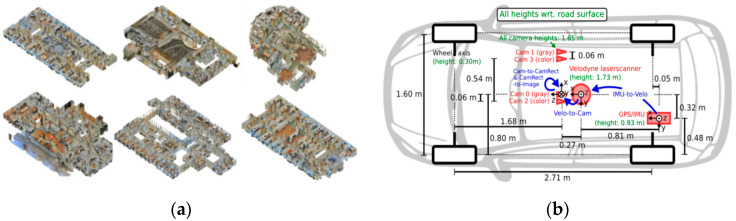
Public datasets: (**a**) S3DIS datasets; and (**b**) KITTI datasets.

(1) The S3DIS contains 3D scanned point clouds of 6 interior regions, with a total of 271 point cloud rooms; each point belongs to one of 13 semantic categories, such as wood panels, bookshelves, chairs, ceilings, and other miscellaneous objects. There are 19,898 point cloud files in the training set and 12 rooms in each region in the test set, for a total of 48 rooms. The data are shown in Figure 7a. At the same time, we also used the public outdoor datasets nuScenes for the evaluation;

(2) At present, the largest international dataset for autonomous driving situations is KITTI. With up to 15 vehicles and 30 pedestrians per image, as well as varying degrees of occlusion and truncation, KITTI comprises real image data gathered from urban, rural, and highway situations. It can be used for target detection and data multimodality studies. The data acquisition is shown in Figure 7b;

(3) SSKIT is a bimodal semantic segmentation dataset obtained by registration and annotation using KITTI data in this paper. SSKIT contains point clouds and image data of 984 outdoor urban scenes with 9 semantic categories, such as pedestrian, car, bicycle, road, background, etc. There are 778 scene samples in the training set and 206 scene data in the validation set. To obtain better semantic segmentation data and to better validate the effectiveness of this representation, KITTI data were registered and labeled. The data were roughly divided into 12 scenes according to scenes, such as city walking street, forest road, other scenes, pedestrian-only compounds, and city highway. We selected appropriate training and testing sets in different scenarios to prevent data imbalances from affecting the experimental results.

**Figure 8:**
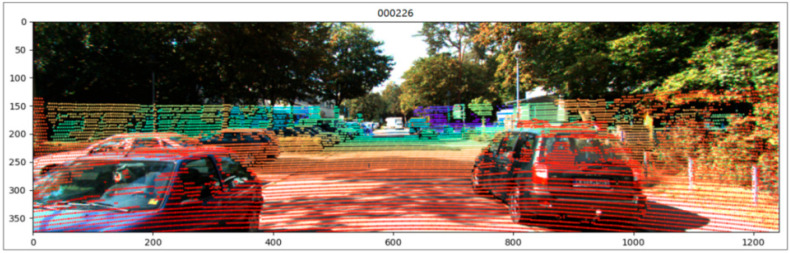
3D point clouds projection to 2D image.

The SSKIT registration and labeling process is as follows:

Using the image, the point clouds, and the camera’s parameter matrix, the point cloud data are projected into the 2D image, and the reflectance value is changed to one. The data are expressed as a vector and the 3D point cloud data $x_{i}$ are projected into the 2D image to obtain ${y^{\prime }}_{i}$. The projection uses the left color camera projection matrix $P_{rect}^{2}$ and the left laser to the camera coordinate system transformation matrix $T_{2}$. Figure 8 shows the effect of the projection on the 2D image. The transformation matrix is shown in the Equation:

(13) $${y^{\prime }}_{i}=P_{rect}^{2}R_{rect}^{0}T_{2}x_{i}.$$

**Figure 9:**
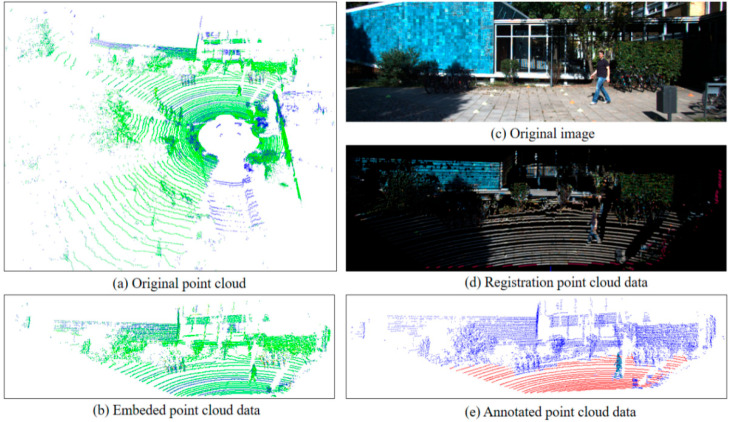
Presentation of data for each phase: (**a**) original point clouds; (**b**) embedded point cloud data; (**c**) original image; (**d**) registration point cloud data; and (**e**) annotated point cloud data.

The point clouds without point-by-point labeling are cropped to obtain 3D data of the same general size as the image data, and all information on the road surface is labeled. The visualization of the points in the data processing is shown in Figure 9.

**Figure 10:**
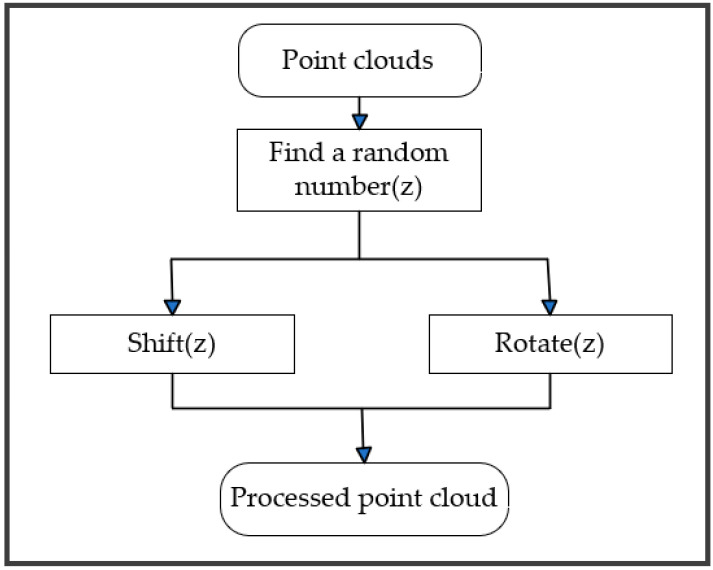
Data enhancement module.

Data Enhancement. In general, the more sufficient the number of samples in the training data, the greater the model’s capacity for generalization and the better the trained model effect. In order to better identify the scene data, a data enhancement code module was added to the dataset in this paper. There are two types of data augmentation, one is for the S3DIS dataset, which rotates the data randomly around the z-axis and keeps the z-axis unchanged. Because objects such as tables and chairs can reduce the recognition effect after being flipped up and down, using the data enhancement method that keeps the z-axis constant can better train the data. The second data augmentation type is for the small point cloud scene dataset produced in this paper, and the data are shifted randomly in the x, y, and z axes to obtain more training data. As only one frame of the data is used, rotation does not apply to this dataset. Keeping the randomly panned data to data augmentation, the training can better learn the features. A schematic of the data enhancement module is shown in Figure 10.

#### 4.1.2. Experimental Environment

Hardware environment: We use an Intel CORE i7 CPU(Integrated Electronics Corporation) with 12G, NVIDIA GeForce GTX 2080(NVIDIA Corporation) for computing, CUDA11.1, and CuDnn11.1 for the GPU acceleration environment.

Software environment: We use Windows 10 OS with Python 3.7 + Pytorch 1.7.

### 4.2. Registration Experiments

The single-frame data acquired by laser radar are sparse, and more dense data are obtained by registering two consecutive frames of the point cloud. For the problem of unclear edges of many objects, the addition of texture information helps to distinguish the objects. In this experiment, lidar data and color image data were collected separately, registered, and fused. The two frames of point clouds are registered, and the superimposed registered data are then fused with the color images to form a dense color 3D point cloud.

**Figure 11:**
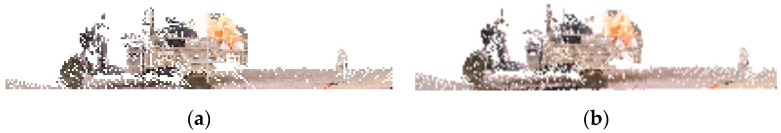
Comparison of data-fusion results: (**a**) the image shows the fusion effect before point cloud alignment; and (**b**) the image shows the fusion effect after point cloud alignment.

As shown in Figure 11, the results of the fusion of the two acquisitions were compared with the results of the fusion of the single-acquisition data, and it was found that the registered data were clearer.

As shown in Table 1, before the registration of point cloud data, the number of colored point clouds in the scene is 3054. After the registration and fusion of 2D and 3D data, the number of colored point clouds is 5427. The aligned data have more complete spatial information.

### 4.3. Indoor and Outdoor Scene Segmentation Experiment

In this paper, experimental validation was performed on the SSKIT dataset and the S3DIS dataset, and the point cloud CNN deep learning network and graph convolutional network were selected for experimental comparison, respectively.

#### 4.3.1. Experimental Results

The experimental results using the SSKIT dataset on PointNet++ [16], DGCNN [32], and the model in this paper are shown in Table 2. The experimental results show that the model in this paper is more accurate and has a high MIoU bias.

First, it should be noted that, after the fusion of the point cloud and two-dimensional image, the data have six dimensions: x, y, z, R, G, B. The term NoRGB means that the R, G, and B columns of the fused data are hidden. The experiments were conducted using the S3DIS dataset and compared with the 3D point cloud semantic segmentation network models of PointNet++ [16], KVGCN [33], and Point Transformer [34]. The experimental results are shown in Table 3. The proposed model has more prominent improvement, and the OAcc of the FGCN is 4.18% higher than that of the KVGCN [33], which is a graph convolution network with better experimental results. The OAcc of the FGCN is 9.38% higher than that of PointNet++ [16], which is a stable CNN network, and 1.38% higher than that of Point Transformer [34], which is a relatively new network. The MIoU results in this paper were 17.65% higher than KVGCN [33], 27.75% higher than PointNet++ [16], and 5.05% higher than Point Transformer [34], which indicates that the proposed model shows a stable improvement in all the evaluation criteria.

As can be seen in Table 4, the accuracy of the FGCN is the highest in some test categories, with more prominent improvements, especially in the challenging categories of the clutter, window, door, column, beam, and column segmentation. Among the categories where other models have lower segmentation results, the window reaches 85.3%, the board reaches 76.1, the column reaches 79.7%, and the door reaches 84.2%. The beam has an extremely low MIoU in other network models, and the model in this paper reaches 73.3%. The accuracy rate of the bookcase category is lower than that of PointTransformer [34], but it is also higher than that of the other networks. We hypothesize that the reason behind the subpar segmentation results for these home furnishings is the uneven distribution of tables, chairs, and sofas within the S3DIS dataset. During the model training process, there are a relatively limited amount of data available for this specific furniture category, leading to inadequate feature learning by the model.

Furthermore, we conducted experiments using the nuScenes outdoor dataset and compared these with the 3D point cloud semantic segmentation network models of Cylin-der3D [14], Cylinder3D++ [14], and SPVCNN++ [40]. The experimental results are shown in Table 5. Our findings indicate that the FGCN outperforms the most advanced models, particularly in transportation categories such as cars, public bicycles, and trucks. Specifically, FGCN achieved outstanding results in these categories, with buses achieving an accuracy of 93.5%, cars an accuracy of 92.6%, motorcycles an accuracy of 85.2%, and trucks an accuracy of 78.2%. While certain metrics may show slightly lower values compared to other networks, the overall performance of the FGCN remains at a high level.

#### 4.3.2. Visualization Display

**Figure 12:**
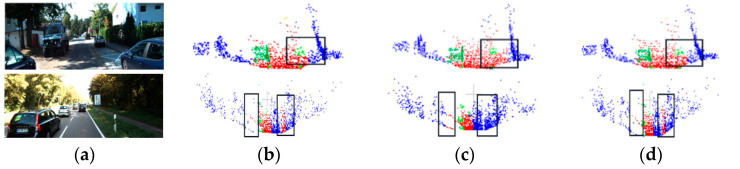
Visualization results of SSKIT with color information: (**a**) original image; (**b**) visualization of DGCNN; (**c**) visualization of PointNet++; and (**d**) visualization of FGCN.

Two scene segmentation results were selected from the SSKIT dataset for visualization and analysis. Figure 12 illustrates how the model used in this study has a superior segmentation effect on the specifics of various road and pavement objects.

**Figure 13:**
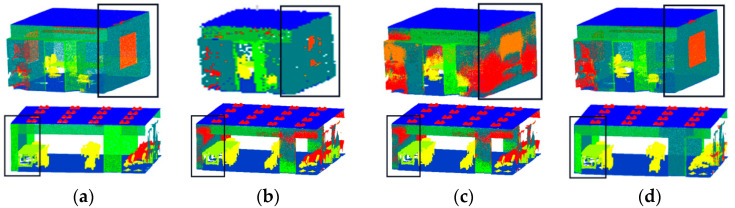
Visualization results of S3DIS: (**a**) visualization of point clouds with original labels; (**b**) visualization of DGCNN; (**c**) visualization of PointNet++; and (**d**) visualization of FGCN.

Figure 13 displays the outcomes of the two rooms of the S3DIS dataset that the model selected for visualization. We can observe from the visualization results that the proposed model has an excellent effect on segmenting windows, walls, and doors, etc.

## 5. Discussion

### 5.1. RGB Feature Set Analysis

In order to test the effect of different texture information feature sets on the experimental results, an experimental comparison between the RGB feature set and the chroma value feature set on the S3DIS dataset was conducted.

**Figure 14:**
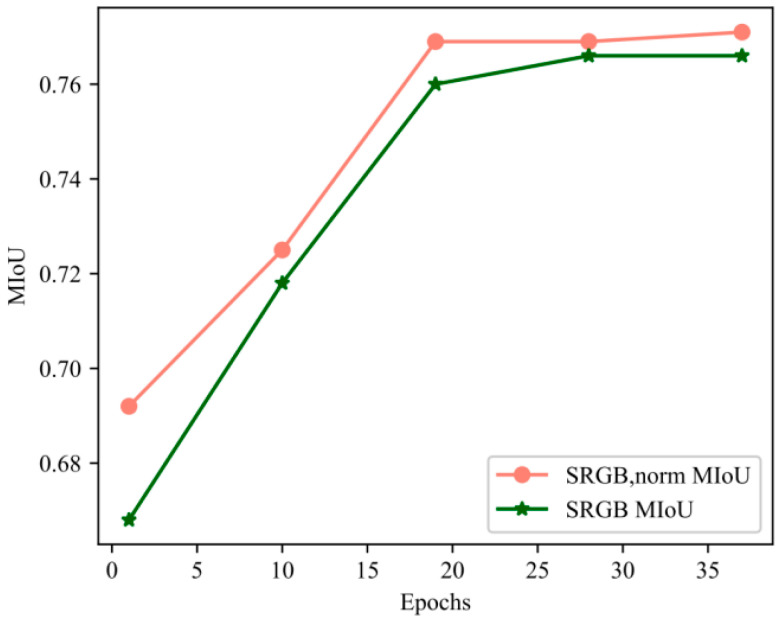
Training curve comparison.

As shown in Figure 14, the training speed was improved for the chroma value feature set $S_{RGB}$ compared to the RGB feature set $S_{RGB,norm}$. There was also an improvement in the training accuracy after processing, as shown in Table 6.

### 5.2. The Effect of Dual-Channel KNN on Speed

**Figure 15:**
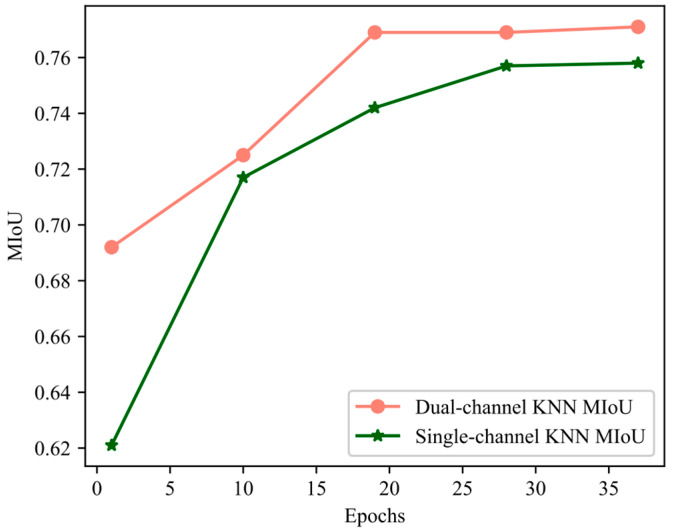
Training curve of MIoU.

The two-channel KNN can extract local features more comprehensively. As shown in Figure 15, the two-channel KNN made the network reach the optimal state more quickly. At the 14th epoch, the MIoU value of the dual-channel KNN reached the value of the single-channel KNN at the 35th epoch.

### 5.3. Robustness Analysis

To test the robustness [45,46] of the model, Gaussian noise [47] was added to some files of the semantic segmentation dataset for testing.

**Figure 16:**
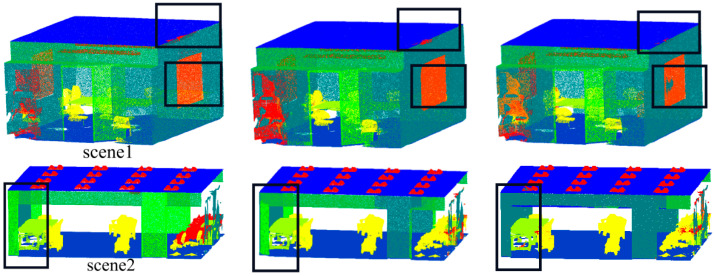

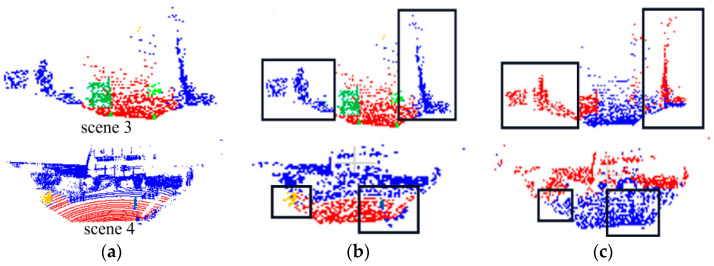
Adding Gaussian noise of SSKIT where: (**a**) is the real label visualization; (**b**) is the model test visualization in this paper; and (**c**) is the visualization of the model in this paper under the data with the addition of Gaussian noise.

As shown in Table 7, after adding Gaussian noise, the segmentation effect of indoor scene 1 and scene 2 was comparable to that before the noise was added. This indicates the greater robustness of our model on the S3DIS indoor dataset. The segmentation effect of outdoor scenes 3 and 4 decreased. The visualization comparison result of scene 3 in Figure 16 demonstrates that despite the accuracy being reduced, the road surface is almost completely segmented, and some unlabeled background information is segmented into means of transportation such as cars. But in scene 4, the leftmost parked bicycle is not segmented, and the people on the road are not segmented. The reason for this is that the point clouds of these scenes are sparse, and the accuracy was reduced due to the noise effect after adding Gaussian noise.

To evaluate the performance of our model in scenarios with sparse point clouds, we downsampled the data in scene 1 from its original density to 1/2, 1/4, 1/6, and 1/8.

After analysis, we discovered that when the point cloud is dense, Gaussian noise has a minimal impact on the segmentation results. However, in cases where the point cloud is sparse, the introduction of Gaussian noise significantly disrupts the segmentation results and can even lead to scene failure, as shown in Table 8. In summary, it is evident that the presence of Gaussian noise reduces the segmentation performance of our model, particularly in outdoor environments with sparse point clouds. This indicates a certain level of sensitivity to noise, resulting in decreased robustness.

In addition, we discussed potential model failures, which include the following:

(1) Through our experiments, we identified a scenario in which our network encounters difficulties. When objects in the scene are severely occluded, our network struggles to accurately identify the edges of these occluded objects, leading to inaccurate segmentation results. To address this issue, we devised our own solution. In future work, we plan to shift from the traditional perspective to the Bird’s-Eye View (BEV) space. The BEV space eliminates the occlusion problem, allowing us to verify the generalization capability of the FGCN model;

(2) When there are significant changes in light intensity, it can impact the 2D image, affecting the fusion result of the image and the point cloud, resulting in reduced segmentation efficiency. However, it is important to note that the model can still operate under these conditions and will not completely fail.

### 5.4. Ablation Studies

The role of each module in the FGCN was demonstrated through ablation experiments. Table 9 shows the experimental results under the parameters of a batch size of 8 and an epoch of 40. We used the grid search method to determine the parameters. After multiple sets of verifications, we found that a batch size of 8 and an epoch of 40 is the best combination.

We chose the S3DIS dataset for evaluation and trained four different models to validate the effectiveness of data enhancement, dual-channel KNN, and spatial attention. The first model excluded the spatial attention module, the second model excluded the dual-channel module, the third model excluded the data augmentation module, and the fourth model included all three components of the FGCN. This set of experiments is denoted as Exp.0. Additionally, to assess the impact of our design on model generalization performance, we conducted an ablation experiment labeled as Exp.1. Exp.1 also involved training four models, and we used the SSKIT dataset for these experiments. The aim of Exp.1 was to compare the experiment with Exp.0 and evaluate the generalization capability of the FGCN network across different environments. Through these two sets of experiments, it was evident that the three modules of the FGCN contribute significantly to improving experimental results, affirming the generalization ability of the FGCN model. It is noteworthy that the accuracy decreased the most when the spatial attention mechanism was not used. This can indicate that the spatial attention module is the key to improving accuracy.

Furthermore, we also considered the aspect of statistical significance. In this context, we refer to the hypothesis being tested as the null hypothesis, which states that ‘there is no significant difference between our method and the missing module methods. At the significance level of α = 0.05, if *p* > 0.05, we accept the null hypothesis, and if *p* < 0.05, we reject the null hypothesis. When comparing our method with the missing module methods on both the SSKIT and S3DIS datasets, the *p*-values were all less than 0.05, leading us to reject the null hypothesis. Consequently, we conclude that there is a significant difference between our method and the missing module methods.

## 6. Conclusions

In this paper, we proposed the FGCN network model that combines 3D point clouds and 2D image color information for feature learning. First, we studied the representation of 2D images and 3D point clouds. The local feature map was obtained by constructing an image-guided topological map structure using multi-channel map convolution. Second, we added a spatial attention mechanism to process the important parts in space to boost the effectiveness of feature learning. Finally, multi-scale feature fusion was used to learn global features, and MLP and log_softmax were used to enhance model stability.

In order to validate the proposed model, we performed an experimental validation on the S3DIS dataset, comparing the FGCN with popular networks. We demonstrated that the FGCN has some advantages for indoor semantic segmentation tasks. On the SSKIT and KITTI datasets, we performed experimental validation to show that the addition of color information to the point clouds has a positive effect on experimental outcomes for outdoor datasets. The experiments showed that the FGCN can perform feature extraction better than the other networks. Nevertheless, the FGCN faces the challenge of low efficiency when extracting neighbor node information during the two-channel graph convolution feature extraction process. Moreover, there is a need for further research on how to enhance the integration of the graph convolution and attention mechanism for improved performance. Graph-focused convolutional networks have great advantages in dealing with unstructured data. Therefore, our future research will focus on efficient feature extraction using graph structures. Simultaneously, we observed that the FGCN tends to fail when the measured object is significantly obstructed. In future work, we plan to shift our focus to the semantic segmentation of the Bird’s-Eye View (BEV) space, where the occlusion problem can be largely mitigated.

## Figures and Tables

**Table 1:** Number of color point clouds in the scene.

	Pre-Registration	Post-Registration
Number	3054	5427

**Table 2:** Comparison of semantic segmentation OAcc (%) and MIoU (%) on the SSKIT dataset.

Method	MIoU	OAcc
PointNet++ [16]	83.40	92.78
PointNet++ [16] +RGB	85.49	93.20
DGCNN [32]	86.40	93.54
DGCNN [32] +RGB	87.22	93.55
Ours+NoRGB	86.70	**94.11**
Ours	**88.06**	93.72

**Table 3:** Comparison of MAcc (%), MIoU (%), and OAcc (%) on the S3DIS dataset.

Method	MAcc	MIoU	OAcc
PointNet [15]	66.20	47.60	78.50
PointNet++ [16]	66.85	50.80	82.20
DGCNN [32]	-	56.10	84.10
KVGCN [33]	72.30	60.90	87.40
Point Transformer [34]	81.90	73.50	90.20
Segcloud [35]	57.35	48.92	80.80
SPG [36]	64.40	54.10	82.90
ShellNet [37]	-	66.80	-
SPH3D-GCN [38]	67.06	57.20	86.94
PCT [39]	67.65	61.33	-
**Ours**	**87.50**	**78.55**	**91.58**

**Table 4:** IoU (%) for selected categories in the S3DIS dataset.

Method	PointNet [15]	PointNet++ [16]	KVGCN [33]	Point Transformer [34]	Segcloud [35]	SPG [36]	SPH3D- GCN [38]	PCT [39]	Ours
ceiling	88.6	93.1	94.5	94.0	90.1	89.9	92.3	92.54	**96.3**
floor	97.3	97.6	94.1	**98.5**	96.1	95.1	98.2	98.42	98.3
wall	69.8	77.2	79.5	86.3	69.9	72.0	71.9	80.62	**87.0**
beam	0.05	00.0	53.4	0.0	0.0	62.8	0.03	0.00	**73.3**
column	3.92	16.2	36.3	38.0	18.4	47.1	17.6	19.37	**79.7**
window	46.3	66.1	56.8	63.4	38.4	55.3	46.8	61.64	**85.3**
door	10.8	58.1	63.2	74.3	23.1	60.0	43.8	48.00	**82.4**
table	52.6	62.3	64.3	**89.1**	70.4	69.2	71.0	85.20	73.5
chair	58.9	65.7	67.5	**82.4**	78.6	73.5	79.7	76.58	78.0
sofa	5.9	35.1	54.3	**74.3**	40.9	45.9	50.3	67.71	55.9
bookcase	40.3	45.2	23.6	**80.2**	58.4	3.2	32.0	46.22	68.8
board	26.4	50.4	43.1	76.0	13.0	8.7	25.8	67.93	**76.1**
clutter	33.2	50.4	53.2	59.3	41.1	52.9	52.7	52.29	**65.5**

**Table 5:** IoU (%) for selected categories in the nuScenes dataset.

Method	Cylinder3D [14]	Cylinder3D++ [14]	SPVNAS [40]	SPVCNN++ [40]	AMVNet [41]	PolarNet [42]	JS3C-Net [43]	2D3DNet [44]	Ours
barrier	82.8	82.8	80	**86.4**	80.6	72.2	80.1	83	83.1
bicycle	29.8	33.9	30	43.1	32	16.8	26.2	**59.4**	35.9
bus	84.3	84.3	91.9	91.9	81.7	77	87.8	88	**93.5**
car	89.4	89.4	90.8	92.2	88.9	86.5	84.5	85.1	**92.6**
construction_vehicle	63	69.6	64.7	75.9	67.1	51.1	55.2	63.7	**77.8**
motorcycle	79.3	79.4	79	75.7	84.3	69.7	72.6	84.4	**85.2**
pedestrian	77.2	77.3	75.6	**83.4**	76.1	64.8	71.3	82	76.5
traffic_cone	73.4	73.4	70.9	77.3	73.5	54.1	66.3	76	**78.6**
trailer	84.6	84.6	81	86.8	84.9	69.7	76.8	84.8	**88**
truck	69.1	69.4	74.6	77.4	67.3	63.5	71.2	71.9	**78.2**
driveable_surface	**97.7**	**97.7**	97.4	**97.7**	97.5	96.6	96.8	96.9	97.1
other_flat	70.2	70.2	69.2	71.2	67.4	67.1	64.5	67.4	**73.2**
sidewalk	80.3	80.3	80	**81.1**	79.4	77.7	76.9	79.8	80.6
terrain	75.5	75.5	76.1	**77.2**	75.5	72.1	74.1	76	76.8
manmade	90.4	90.4	89.3	91.7	91.5	87.1	87.5	92.1	**93.5**
vegetation	87.6	87.6	87.1	89	88.7	84.5	86.1	**89.2**	88.5

**Table 6:** MIoU for selected categories in the S3DIS dataset (%).

Method	MIoU	OAcc
S_RGB_	87.44	91.34
S_RGB,norm_	**87.50**	**91.58**

**Table 7:** Adding Gaussian noise test MIoU (%) comparison.

Scene	Ours	Adding Gaussian Noise
1	71.18	71.70
2	84.15	84.05
3	46.82	44.26
4	72.03	67.88

**Table 8:** Adding Gaussian noise after downsampling to test MIoU (%) comparison.

Sampling Ratio	Ours	Adding Gaussian Noise
1/2	70.65	70.32
1/4	71.05	70.95
1/6	66.34	50.85
1/8	63.88	30.88

**Table 9:** Ablation experiments on S3DIS dataset and SSKIT dataset (%).Y means this module is included in this experiment, N means this module is not included.

Exp	Data Enhancement	Dual-Channel KNN	Spatial Attention	MAcc (%)	MIoU (%)	OAcc (%)
	Y	Y	N	83.33	72.98	89.46
Exp.0	Y	N	Y	85.64	73.65	90.27
	N	Y	Y	87.23	75.32	90.35
	Y	Y	Y	**87.50**	**78.55**	**91.58**
	Y	Y	N	84.48	73.25	89.54
Exp.1	Y	N	Y	85.98	74.23	90.88
	N	Y	Y	87.47	76.47	90.48
	Y	Y	Y	**89.23**	**79.54**	**91.45**

## Data Availability

The dataset related to this article can be found at: https://goo.gl/forms/4SoGp4KtH1jfRqEj2 (accessed on 28 September 2023) https://www.cvlibs.net/datasets/kitti/eval_object.php?obj_benchmark=3d (accessed on 28 September 2023).

## Abbreviations

The following abbreviations are used in this manuscript:

FGCN	Fusion Graph Convolutional Network
KNN	K-Nearest Neighbors
GCN	Graph Convolutional Network
MLP	Multi-Layer Perception
SSKIT	Semantic Segmentation KITTI
OAcc	Overall accuracy
MAcc	Mean Accuracy
BEV	Bird’s-Eye View
MIoU	Mean Intersection over Union
